# An Embodied Brain Model of the Human Foetus

**DOI:** 10.1038/srep27893

**Published:** 2016-06-15

**Authors:** Yasunori Yamada, Hoshinori Kanazawa, Sho Iwasaki, Yuki Tsukahara, Osuke Iwata, Shigehito Yamada, Yasuo Kuniyoshi

**Affiliations:** 1 Graduate School of Information Science and Technology, The University of Tokyo, Tokyo, Japan; 2 Department of Paediatrics and Child Health, Kurume University School of Medicine, Fukuoka, Japan; 3 Congenital Anomaly Research Center, Kyoto University Graduate School of Medicine, Kyoto, Japan

## Abstract

Cortical learning via sensorimotor experiences evoked by bodily movements begins as early as the foetal period. However, the learning mechanisms by which sensorimotor experiences guide cortical learning remain unknown owing to technical and ethical difficulties. To bridge this gap, we present an embodied brain model of a human foetus as a coupled brain-body-environment system by integrating anatomical/physiological data. Using this model, we show how intrauterine sensorimotor experiences related to bodily movements induce specific statistical regularities in somatosensory feedback that facilitate cortical learning of body representations and subsequent visual-somatosensory integration. We also show how extrauterine sensorimotor experiences affect these processes. Our embodied brain model can provide a novel computational approach to the mechanistic understanding of cortical learning based on sensorimotor experiences mediated by complex interactions between the body, environment and nervous system.

Human foetuses generate various types of movements and sense their own body and environment^1^,^2^. Spontaneous movements and somatosensory responses can be observed as early as 8 weeks^1^,^3^. Ultrasound examinations of foetuses and empirical studies of newborns have revealed sophisticated behaviours and cognitive repertoires, indicating that foetuses begin learning through sensorimotor experiences inside the uterus^4^,^5^.

Early neural development based on sensorimotor experiences has been examined in newborn animals and preterm humans. In the spinal cord, somatosensory feedback related to spontaneous movements has been shown to be instrumental for the formation of reflex sensorimotor circuits^6^,^7^,^8^. From the third trimester of pregnancy, afferent sensory feedback is inputted to the cortex through thalamocortical connections, which guides the patterning of cortical connectivity and the formation of sensory response properties^9^. In the somatosensory system, imaging studies have indicated the important role of sensory feedback that results from spontaneous foetal movements in the establishment of body representations in the somatosensory cortex^10^,^11^. Despite the accumulating evidence supporting the importance of sensorimotor experience in neural development as early as the foetal period, the learning mechanisms by which intrauterine sensorimotor experiences guide cortical learning, including the factors in prenatal experiences that are needed for normal development, remain unclear. However, the investigation of this causal link between sensorimotor experiences and cortical learning is particularly challenging in human foetuses owing to the technical and ethical difficulties associated with experiments related to overall interactions between the brain, body and environment (‘embodied interactions’).

Brain models have been actively developed to aid our understanding of neuronal mechanisms^12^. Whereas some studies have focused on how normal intrinsic cortical activities emerge from large-scale brain models with biologically detailed structures and properties^13^, others have investigated learning mechanisms using comparatively simple brain models with small numbers of neurons^14^. However, simulating the brain alone does not address the underlying neuronal mechanisms by which sensorimotor experiences guide cortical learning via embodied interactions. Because embodied interactions induce specific statistical regularities in sensory feedback that influence neural dynamics and learning^15^,^16^, the manner in which embodied interactions shape sensorimotor experiences is critical when investigating the mechanisms of cortical learning.

In this paper, we present a novel, integral embodied brain model based on anatomical and physiological data that incorporates a cortex, spinal circuit and musculoskeletal body with sensory receptors for proprioception, tactile perception and vision within a model of a uterine environment. Using this model, we simulated cortical learning based on activity-dependent plasticity through interactions between the brain, body and environment related to spontaneous bodily movements. The outcomes of different somatosensory conditions in intrauterine versus extrauterine environments were also compared. The detailed investigations of the learned sensory response properties and the information structures of sensory feedback demonstrated that intrauterine sensorimotor experiences can facilitate the cortical learning of body representation and subsequent visual-somatosensory integration and how extrauterine sensorimotor experiences affect these processes. Based on these experiments, we demonstrate that the embodied brain model enables us to explore the causal link between sensorimotor experiences and cortical learning.

## Results

### Model overview

**Figure 1:**
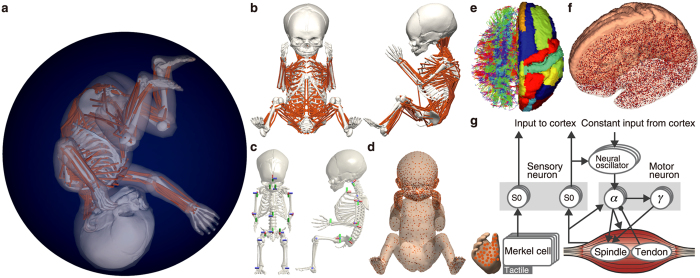
Overview of the embodied brain model of a human foetus. (**a**) Foetal body model in the intrauterine condition. (**b**) Foetal musculoskeletal model. Each line is a uniaxial muscle actuator in the body. **(c)** Joint positions and directions. The cylinders represent the joint axes. (**d**) Tactile sensors throughout the body. The distribution was based on human two-point discrimination data. **(e)** Representative tractography and parcellation of preterm infant brain MRI scans. The colours on the left indicate the spatial relationships between the fibre end points. Red: left-right; blue: dorsal-ventral; green: anterior-posterior orientations. The colours on the right represent the brain regions defined in the brain atlas. **(f)** Model neurons in the cortical model. The dots represent neurons. **(g**) Spinal circuit model. The arrows and filled circles represent the excitatory and inhibitory connections, respectively. S0 represents sensory interneurons.

Here, we provide an overview of the basic properties of our model (Fig. 1). A full description is provided in the Methods and Supplementary Methods.

We constructed a foetal musculoskeletal body model with size, weight and muscle force parameters that correspond to 32 weeks of gestation by incorporating the following data sets: (i) magnetic resonance imaging (MRI) of historical specimens of a human foetus (Supplementary Fig. 1); (ii) computed tomography scan data from a foetal skeleton replica; and (iii) experimental data related to muscle dynamics, proprioception and tactile perception. The foetal model simulates 21 rigid body parts connected by 20 joints with 36 degrees of freedom, 390 muscles with proprioceptive receptors and 3,000 tactile mechanoreceptor models in the entire body (Fig. 1a–d, Supplementary Tables 1 and 2). We also modelled vision as a camera model fixed in the head. The foetal model was placed inside a uterine environment model consisting of a uterine membrane and amniotic fluid.

The neural model consists of a spinal circuit model and a cortical model (Fig. 1e–g). The spinal circuit model generates muscle commands with basic neuromuscular loops and relays sensory feedback related to proprioception and tactile perception to the cortical model. This spinal circuit has neural oscillators, α and γ motor neurons, and sensory interneurons that are based on experimental data^17^.

The cortical model includes 2.6 million spiking neurons and 5.3 billion synaptic connections. The cortical connectivity and axonal conductance delays were determined based on MRI and diffusion tensor imaging extracted from 15 preterm human infants. The cell bodies of the model neurons were also allocated according to the voxels of the grey matter surface extracted from the MRI data (30 neurons/voxel). We input the somatosensory and visual signals to the neurons of each area of the primary sensory cortex. The spiking dynamics of each neuron were simulated with a leaky integrate-and-fire neural model with conductance-based excitatory and inhibitory synapses. Activity-dependent plasticity was modelled with a spike-timing-dependent plasticity (STDP) rule based on observations of the developing neocortex^18^,^19^.

### Whole-body movements and intrinsic cortical activities

Although each integral part of our model is based on anatomical and physiological data, it is important to confirm the biological relevance of the overall behaviours when they are combined. To this end, we investigated whether our model reproduced the normal properties of whole-body spontaneous movements and intrinsic cortical activity patterns that have been reported in biological studies.

Bodily movement patterns constrain sensorimotor experiences and consequently influence the statistical regularities in sensory feedback that affect neural learning. Thus, the evaluation of whether simulated movements can capture the features of human foetal movements is important for the investigation of cortical learning based on sensorimotor experiences. Whole-body spontaneous movements are commonly observed in foetuses and infants^20^,^21^,^22^, and their properties are quantitatively and qualitatively characterized for the clinical purposes of predicting subsequent development^21^,^22^.

We investigated simulated movements in terms of these properties. We introduced random inputs to the motor neurons of the foetal model inside the uterus at the beginning of the simulation. After this procedure, the model generated self-sustained whole-body movements in a closed-loop manner using proprioceptive sensory feedback (Supplementary Video 1). We analysed these movements and confirmed that the simulated movements exhibited normal behavioural features that have been reported in human studies in terms of the following aspects (Supplementary Fig. 2 and Supplementary Methods): the participation of multiple body parts^21^,^22^ (Supplementary Fig. 2a); continuously varying combinations of body part movement directions^21^,^22^ (Supplementary Fig. 2a); well-coordinated and self-organized patterns^22^,^23^ (Supplementary Fig. 2f); and chaotic and fractal properties in limb movements^24^,^25^ (Supplementary Fig. 2c–e).

We also evaluated the dynamic properties of the cortical model in terms of intrinsic activity in the resting state, which is one of the most frequently investigated brain activities across multiple anatomical scales. Human studies have reported structure-function relationships of connectivity across cortical regions^26^. Recently, these relationships have been assessed in human neonates and preterm infants^27^. In addition to these relationships, we also confirmed the biological relevance of the simulated intrinsic cortical activity in terms of several representative features that have been reported in *in vivo* animal studies^28^,^29^,^30^,^31^,^32^.

**Figure 2:**
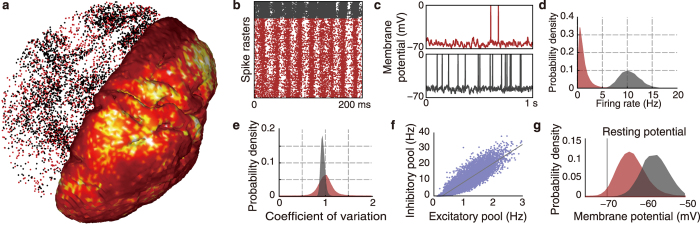
Simulated intrinsic cortical activity and the properties of the neuronal firing activities. (**a**) A snapshot of the activities. On the left, the red (black) dots indicate the spikes of the excitatory (inhibitory) neurons. On the right, the colours represent the mean firing rates. **(b)** Spike rasters from one brain area. **(c)** Example time courses of the membrane potentials of the excitatory and inhibitory neurons. **(d)** Firing rate distributions. The mean firing rates of the excitatory neurons were low (1.2 Hz). The distributions of the firing rates conformed to lognormal distributions (*R*^2^ = 0.97 for excitatory; *R*^2^ = 0.98 for inhibitory; *P* < 10^−20^). **(e)** Coefficient of variation of the inter-spike intervals. The averages were 1.00 and 0.95 for the excitatory and inhibitory neurons, respectively, which indicated that the spike trains were highly irregular and followed a Poisson distribution. **(f)** Relationship of the firing rates between the excitatory and inhibitory neurons (*r* = 0.86; *P* < 10^−20^). **(g)** Membrane potential distributions. The average membrane potentials were −64.3 mV and −57.6 mV for single excitatory and inhibitory neurons, respectively. These average potentials were high compared with the resting potentials (−70 mV). **(b–e,g)** Red and black represent the excitatory and inhibitory neurons, respectively.

Using fixed cortical connectivity weights, we simulated intrinsic cortical activity under conditions lacking any sensory feedback or noise. To trigger the cortical activity, we initially introduced Poisson spikes with a frequency of 1 Hz to all neurons for 100 ms. The cortical model then exhibited autonomous, self-sustained activities with no external inputs. We analysed these cortical activities for 60 s. We confirmed that the model exhibited self-sustained activities that included the following distinctive properties that have been observed in physiological cortical activity (Fig. 2, Supplementary Fig. 3 and Video 1): low firing rates of individual neurons that approximate lognormal distributions^30^ (Fig. 2d); irregular neuronal firing following a Poisson distribution^31^ (Fig. 2e); a network balance between excitation and inhibition^29^ (Fig. 2f); greater depolarizations of the average membrane potentials relative to the resting potentials^32^ (Fig. 2g); correlations between structural and functional connectivity across cortical regions^26^,^27^ (Supplementary Fig. 3a); and responsiveness to single spikes^28^ (Supplementary Fig. 3b–e).

For both the whole-body movements and intrinsic cortical activities, the biologically plausible dynamic properties were not directly built in, but rather autonomously emerged from the overall interactions between the model components based on anatomical and physiological data.

### Learning experiment setup

**Figure 3:**
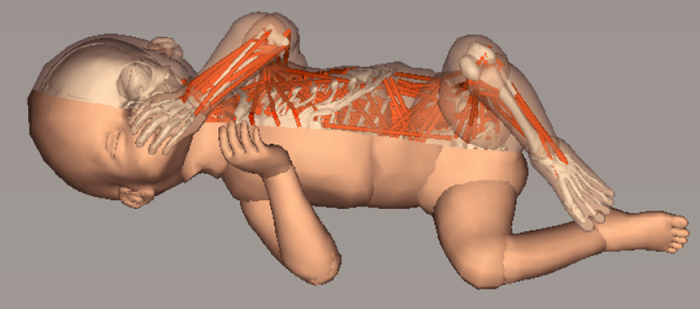
Extrauterine environmental condition. The body model was laid on a flat, rigid bed.

After confirming the relevance of the model with regard to the biological data, we next investigated how intrauterine sensorimotor experiences contribute to cortical learning. To this end, we simulated and compared cortical learning via STDP with somatosensory (tactile perception and proprioception) feedback under two environmental conditions, i.e., intrauterine and extrauterine environmental conditions (Figs 1a and 3). The two simulations initiated learning using identical body and nervous models, STDP parameters, and learning time; thus, only the environmental conditions differed between these simulations. In the intrauterine environment, the foetal model received forces from the uterine membrane, amniotic fluid and physical contact between body parts in addition to gravity and buoyancy forces. In contrast, in the extrauterine environment, the model was subject to only the force of gravity and physical contact forces between the body parts and the flat, rigid bed. See the Supplementary Methods for more details.

After learning simulation for 1,000 sec, we fixed the cortical connectivity weights and compared the responses of the learned cortical models to sensory input during intrinsic activity. To investigate the properties of the learned sensory response, we computed the z-scores of the average firing rates relative to those of the intrinsic cortical activities within each voxel. When a z-score exceeded 3, the voxel was considered to significantly respond to the sensory input.

### Cortical learning of body representations

**Figure 4:**
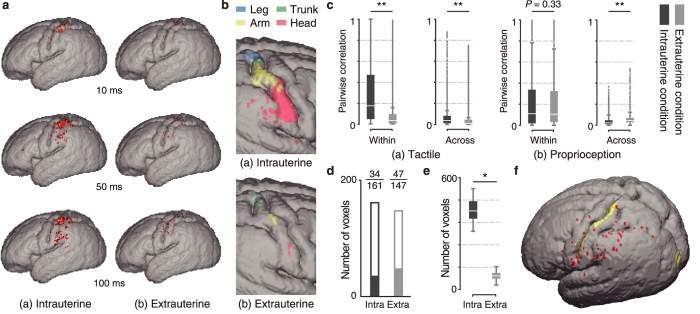
Cortical learning under intrauterine and extrauterine conditions. **(a)** An example of the evolution of the cortical activities over time upon somatosensory input to the right arm. The coloured cortical regions represent the regions that significantly respond to the sensory input. **(b)** Cortical regions that significantly respond to specific single body parts. **(c)** Pair-wise correlation of the sensory feedbacks within and across body parts during simulated whole-body movements (**P < 10^−24^). **(d)** Numbers of voxels that significantly respond to proprioception feedback during whole-body movements. The white and coloured bars indicate voxels that respond to proprioception feedback from specific single body parts and across multiple body parts, respectively. **(e,f**) Cortical regions that significantly respond to simultaneous visual-somatosensory inputs, but not separate inputs. **(e**) The number of these cortical regions significantly increased in the learned cortical model inside the uterus (*P < 10^−3^). **(f)** The red regions are the cortical regions that responded to simultaneous inputs in the model learned inside the uterus. The yellow regions represent the cortical regions that received the sensory input.

We investigated the properties of the responses to somatosensory inputs from the head, arm, trunk and leg. We first investigated how the somatosensory input from each body part propagated over time in the cortical models that had learned under the intrauterine and extrauterine embodied interactions. The somatosensory inputs spread over a wider range of cortical regions in the model learned under the intrauterine condition than the model learned in the extrauterine condition (Fig. 4a).

We also compared the learned body representations by specifically testing the cortical responses to multiple patterns of somatosensory inputs from each body part. Consequently, we identified cortical regions that significantly responded to a single body part in both intrauterine and extrauterine conditions (Fig. 4b). Furthermore, the intrauterine learning significantly increased the number of the cortical regions that responded to specific single body parts compared to the extrauterine learning for all tested body parts (*P* < 10^−3^, rank-sum test; Fig. 4b and Supplementary Fig. 4a). These results indicated that the sensorimotor experiences of the intrauterine embodied interactions are advantageous for the formation of cortical body representations.

### Sensory information structures of the intrauterine and extrauterine embodied interactions

To gain insight into the causes underlying the above differences, we analysed the sensory signals resulting from the intrauterine and extrauterine embodied interactions. We calculated pairwise Pearson’s correlation coefficients for the sensory signals within and across the body parts in each sensory modality. We found that the embodied interaction inside the uterus provided a body part-specific modular organization in both tactile perception and proprioception. The correlation between the tactile signals within body parts exhibited a substantial and significant increase compared with the significant but small increase in the correlation across the body parts (*P* < 10^−24^, rank-sum test; Fig. 4c). Regarding proprioception, although the correlations within the body parts did not differ significantly between the intrauterine and extrauterine conditions (*P* = 0.33, rank-sum test; Fig. 4c), those across the body parts were significantly reduced inside the uterus compared with those outside the uterus (*P* < 10^−24^, rank-sum test; Fig. 4c). Considering the above results, the spatially-continuous pressure exerted by the amniotic fluid resistance and the uterine membrane could be the primary contribution to the increased correlations within the body parts in terms of the tactile sensory feedback. In contrast, regarding proprioception, the increased correlations across the body parts outside the uterus reflected the effects of the movements of each body part on the other body parts; for example, the contact of the limbs with the ground could change the ground reaction force on other body parts. These analyses clearly showed that different environmental conditions induce different statistical regularities in the sensory feedback via embodied interactions.

### Effects of sensory information structures on the learning of body representations

Intrauterine embodied interactions produced a substantial increase in the correlations between the tactile sensors within the body parts and a decrease in the correlations between the proprioception sensors across body parts. To deepen our understanding of the relationship between the sensory information structures and cortical learning, we next analysed the learned sensory properties via the separate input of sensory feedback from each modality.

Intrauterine cortical learning significantly increased the number of cortical regions that responded to each tactile and proprioceptive sensory feedback inputs in all of the tested body parts (*P* < 0.05, rank-sum test; Supplementary Fig. 4b,c). This significant increase in the learned cortical response to tactile sensory feedback was assumed to have resulted from the increased correlations within the body parts in the intrauterine condition. In contrast, the correlations between the proprioceptive sensors within the body parts did not differ between the intrauterine and extrauterine embodied interactions, although the correlations across the body parts significantly decreased in the intrauterine interactions. We further analysed how the decreased correlations across the body parts facilitated the learning of proprioceptive sensory responses to specific body parts. We investigated the ratios between the learned sensory responses across multiple body parts and single body parts. Specifically, we first recorded the proprioceptive signals during whole-body movements. Then, we collected cortical regions that significantly responded to the recorded proprioceptive signals from the entire body (set C1). In parallel, we collected cortical regions that responded to the signals from individual single body parts (set C2) by dividing the recorded signals into individual body part components and feeding them into the cortex model one by one. Finally, we defined C3 = C1 − C2 as the set of cortical regions responding to the input across multiple body parts. The intrauterine embodied interactions significantly decreased the ratios of learned neurons that responded to proprioceptive sensory inputs across multiple body parts (intrauterine condition, 21.1%; extrauterine condition, 32.0%; *P* = 0.031, χ^2^ test; Fig. 4d). This result indicated that the reduced correlations across body parts promote learning about single body parts rather than multiple body parts.

Taken together, these results indicate that the increased correlations within body parts in tactile sensation and the decreased correlations across body parts in proprioception that are evoked by intrauterine embodied interactions facilitate the learning of body representations for specific single body parts.

### Visual-somatosensory integration

Does the learning of body representations inside the uterus facilitate cortical learning (e.g., multisensory integration) during postnatal periods? Developmental studies have indicated that the learning about one’s own body through tactile and proprioceptive perceptions is considered a significant prerequisite for the subsequent development and integration of other senses, such as vision and audition^33^. To examine this hypothesis regarding the visual-somatosensory integration that has been experimentally observed in the parietal cortex^34^, we investigated the cortical regions that significantly responded to simultaneous visual-somatosensory inputs but not to separate inputs. Specifically, we extracted the two cortex models learned with somatosensory feedback under intrauterine and extrauterine conditions and embedded them in neonate body models with parameters appropriate for 40 weeks of gestational age. We generated arm movements in the right arm using motor commands during simulated whole-body movements under the extrauterine condition, in which the body was laid on a flat bed, and investigated cortical responses to the somatosensory and visual feedback.

The results indicated that the number of cortical regions responding to the simultaneous cross-modal inputs significantly increased in the cortical model learned under the intrauterine condition compared with the extrauterine condition (*P* < 10^−3^, rank-sum test; Fig. 4e,f). This result suggests that the learning of body representations via intrauterine embodied interactions provides a foundation for visual-somatosensory integration after birth.

## Discussion

In this study, we present an integral model of the coupled brain-body-environment system based on anatomical/physiological data. To examine the biological relevance of the dynamic properties of the model, we confirmed that the whole-body movements and intrinsic cortical activities match the typical patterns and quantitative indices from biological observations. Using this model, we compared cortical learning via embodied interactions under intrauterine and extrauterine environmental conditions. The experiments demonstrated that intrauterine embodied interactions could be advantageous for guiding the cortical learning of body representations. This observation was due to the body part-specific modular structure of somatosensory feedback characterized by strong correlations within body parts in tactile perception and weak correlations across body parts in proprioception. We also identified the possibility that the cortical learning of body representations facilitated by intrauterine embodied interactions could provide a foundation for the subsequent learning of visual-somatosensory integration during the postnatal period.

Studies across multiple disciplines, such as developmental science, cognitive neuroscience and robotics, have increasingly argued that investigations of embodied interactions can provide insights into human cognition and development^15^,^16^,^35^,^36^,^37^,^38^. Consistent with theories and computational models, some empirical evidence from animal studies supports the notion that sensorimotor experiences via embodied interactions play important roles in early functional neural development^6^,^8^,^10^. However, in human foetuses, technical and ethical difficulties have prevented investigations of the causal link between sensorimotor experiences via embodied interactions and early cortical learning. In the present study, we demonstrated that our embodied brain model can be used to examine this causal link by showing how intrauterine embodied interactions produce a multitude of regularities in sensorimotor experiences and facilitate cortical learning. We also found that the intrauterine embodied interactions provide a better scaffold for the formation of cortical body representations than the extrauterine embodied interactions. Previous experimental studies on animal newborns and preterm human infants have suggested that the formation of body representation begins in the foetal period^10^,^11^. Our results support this notion from a novel perspective that focused on embodied interactions.

In addition, our experiments demonstrated that the cortical learning of body representations via intrauterine embodied interactions can play an important role as a scaffold for visual-somatosensory integration in postnatal periods. This result provides a mechanistic understanding of an incremental developmental process. Several studies have suggested that the development of body representation provides a foundation for the subsequent development of higher cognitive functions and multi-sensory integration^33^,^39^. In developmental science, this incremental process has been recognized as one of the important characteristics for understanding development^35^,^40^. However, the neuronal mechanisms underlying this incremental development have not been investigated in depth. Our computational approach offers a distinct opportunity to explore the mechanisms by which multiple functions emerge during development in an incremental manner.

Neurodevelopmental disorders, such as autism spectrum disorders and attention-deficit hyperactivity disorder, have recently increased in prevalence and have received considerable attention^41^,^42^. Long-term follow-up studies have shown that perinatal environmental factors and diverse genetic factors influence the risk of developmental disorders later in life^42^. Because changing environmental conditions alter sensorimotor experiences, the peculiarities of sensorimotor experiences may have atypical effects on development. Among the perinatal environmental factors, preterm birth has been actively investigated, and the related studies suggest that even preterm infants without apparent brain injuries experience motor, cognitive, and learning difficulties and are at a greater risk of developmental disorders than their term-born counterparts^43^. Additionally, recent functional connectivity studies have reported differences between preterm infants and full-term neonates at term-equivalent ages that suggest that preterm infants follow different trajectories of brain development from those of full-term neonates^44^,^45^; these different trajectories are partially due to the differences in their perinatal experiences^44^,^45^. However, the causal links between the sensorimotor experiences of embodied interactions under specific environmental conditions and abnormal brain development remain unknown. Our model may be useful for examining this causal link and facilitating our understanding of neurodevelopmental disorders. In the present study, we demonstrated that the extrauterine environmental conditions cause immature cortical learning of body representation that subsequently leads to negative effects on multi-sensory integration. These results suggest that abbreviated intrauterine periods and early exposure to extrauterine environments might negatively influence cortical learning in preterm infants. Indeed, clinical studies support our hypothesis by reporting that preterm infants are at high risk for somatosensory processing impairments^33^ and sensory integration dysfunction^46^. Combined with mainstream developmental science, our computational approach may hold promise for exploring the origins of neurodevelopmental disorders by providing insights into how different environmental conditions alter sensorimotor experiences and influence cortical learning.

Our results provide initial insights into the mechanistic link between sensorimotor experiences via embodied interactions and the cortical learning of body representation. Using comparative experiments related to the intrauterine and extrauterine conditions and detailed analyses of sensory feedback, we showed how intrauterine embodied interactions provide body part-specific modular organization to proprioception and tactile sensory feedback and facilitate increased cortical learning of body representations. This mechanistic insight was possible because our computational approach allowed for manipulations and detailed analyses that are difficult or impossible in human studies. For the developmental care of preterm infants in neonatal intensive care units, caregivers often implement positioning or swaddling methods that are designed to provide a sense of containment similar to the intrauterine environment^47^. Experimental studies on animals have shown that in addition to the influence of early exposure to abnormal experiences on neural circuits^6^,^48^, the enrichment of environmental conditions improves neural circuits and subsequent neuronal/behavioural development^49^. However, in the case of preterm human infants, the effect of developmental care on neuronal development remains unexplored, and the developmental benefits are still controversial^50^. As shown in the present study, our embodied brain model can aid in the acquisition of insights into the mechanisms underlying early functional brain development. This model also holds promise for investigating how different environmental conditions and interventions influence sensorimotor experiences and cortical learning. These insights were made possible by the uniqueness of our model, i.e., a unified platform that consisted of the nervous system, body and environment models. From this perspective, our embodied brain model might be useful for improving current developmental care.

In summary, we propose a computational framework to bridge the causal gap between the sensorimotor experiences of embodied interactions and cortical learning. We have shown that our results are in agreement with several experimental biological observations. Finally, we offer novel mechanistic insight into how sensorimotor experiences guide cortical learning. Overall, our embodied brain model could open new avenues for exploring the effects of sensorimotor experiences on cortical learning situated in complex interactions between the body, environment and nervous system.

## Methods

### Extracting cortical connectivity

Brain MRI data for 15 preterm infants (males/females: 9/6, corrected age at MRI: 38.9 ± 1.4 weeks, gestational age at birth: 29.3 ± 2.6 weeks) were used to build the cortical model. Preterm infants without brain lesions who were admitted to the neonatal intensive care unit of Kurume University Hospital were recruited with informed consent from their parents. All subjects were followed until approximately 18 months of corrected age and showed normal cognitive and motor development. They were assessed using the Bayley Scales of Infant and Toddler Development-III (Pearson, London, UK), the Gross Motor Function Classification System^51^, and neurological examinations by an experienced clinical psychotherapist and consultant neonatologists familiar with the neuro-developmental assessment of infants and young children. This study was conducted under the approval of the local ethical committees of Kurume University School of Medicine and the University of Tokyo and performed in accordance with the Ethical Guidelines for Medical and Health Research Involving Human Subjects.

For each subject, diffusion-weighted images of 36 axial slices were acquired with a Signa HDxt 3.0 T (General Electric, Milwaukee, WI, USA) with a single-shot echo-planar imaging sequence using the following parameters: TR (time repetition) = 15,000 ms, TE (time echo) = 86.4 ms, FOV (field of view) = 240 × 240 mm^2^ and resolution = 0.94 × 0.94 × 3 mm^3^. Diffusion gradients with a b-value of 1,000 s/mm^2^ were applied in 7 non-collinear directions. We also acquired a reference scan without a diffusion gradient (*b* = 0). All images were visually inspected before analysis to ensure the absence of apparent or aberrant artefacts.

We first pre-processed all images with a standard pipeline that included reorientation, re-sampling, intensity correction and brain extraction using iBeat^52^ developed for the neonate and infant brain. We next used SPM^53^ to parcellate the brain of each subject into 90 anatomically labelled cortical and subcortical regions in accordance with a brain atlas for neonates^54^ adopted from the Automated Anatomical Labelling Atlas^55^. Diffusion tractography was performed on the diffusion data from each subject using a deterministic diffusion fibre-tracking method implemented in DSI Studio (http://dsi-studio.labsolver.org) with a minimal allowed fractional anisotropy of 0.1 and a maximal turning angle of 70°. We counted the number of fibres passing through each pair of regions and constructed binary connectivity maps for each individual subject. Finally, we integrated the results into a connectivity matrix by extracting the connections that were consistent across subjects. We also calculated the average fibre length for every connected pair to determine the axonal conductance delays between cortical regions.

### Cortical model

We extracted 87,555 voxels of the grey matter surface and allocated 30 cell bodies of model neurons to each voxel. The 30 cell models in each voxel consisted of 25 excitatory and 5 inhibitory neurons. Each neuron received post-synaptic inputs from 1,000 excitatory and inhibitory neurons each. The excitatory post-synaptic inputs were composed of global and local connections at a ratio of 3:7 based on neuroanatomical data^56^. We determined the excitatory global connections using the extracted cortical connectivity between the cortical regions described above. Excitatory local connections and inhibitory connections were randomly generated in adjacent voxels with constant connection probabilities (0.1 for excitatory and 0.5 for inhibitory). We determined the connection distances for the global connections using the extracted average fibre lengths between the cortical regions and connection distances for the local connections using the Manhattan distance between voxels. We randomly set the axonal conductance delays between *d*_0_ −1 and *d*_0_ + 1, where *d*_0_ was determined by the connection distance such that its maximal value was 20 ms.

To simulate neuron dynamics, we used a leaky integrate-and-fire neuron model with conductance-based excitatory (E) and inhibitory (I) synapses described as follows:


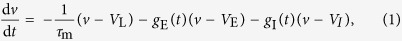



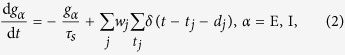


where *v* is the membrane potential, *g*_E_ and *g*_I_ are the excitatory and inhibitory synaptic conductances normalized by the membrane capacitance, respectively, *δ*(*t*) is the delta function, and *w*_*j*_, *d*_*j*_, and *t*_*j*_ are the weight, delay, and spike time of the synaptic input from neuron *j*, respectively. When *v* reached a threshold *V*_thr_ = −50 mV, a spike was emitted and *v* was reset to *V*_reset_ = −60 mV after an absolute refractory period of 2 ms. We also set the membrane time constant to *τ*_m_ = 20 ms for excitatory neurons and *τ*_m_ = 10 ms for inhibitory neurons. The decay constant was set to *τ*_s_ = 2 ms, and the reversal potentials of leak, excitatory and inhibitory postsynaptic currents were set to *V*_*L*_ = −70 mV, *V*_*E*_ = 0 mV and *V*_I_ = −80 mV, respectively. We determined the initial weight distribution for the excitatory-to-excitatory connections such that the amplitude of the excitatory postsynaptic potentials (EPSPs) simulated from the resting potential obeyed a lognormal distribution based on experimental data^32^,^57^. We set the mean and variance of the lognormal distribution to 0.96 mV and 1.2^2^ mV^2^, respectively. We set the weights of the other connections to constant values of *w*_*j*_ = 0.018, 0.002 and 0.0025 for the excitatory-to-inhibitory, inhibitory-to-excitatory and inhibitory-to-inhibitory connections, respectively. The parameters for the neuron models and their weights were based on a previous study^32^.

The excitatory-to-excitatory connections were plastic and changed at every firing event according to a STDP rule^18^. If a spike from an excitatory pre-synaptic neuron *j* arrives shortly before a post-synaptic neuron 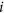 spike, then the synapse *w*_*j,i*_ is potentiated. By contrast, if the spike arrives shortly after the postsynaptic neuron spike, the synapses are depressed. Thus, the STDP rule allows the network to strengthen and learn causal interactions. More specifically, the STDP rule modifies the synaptic weight as *w*_*j,i*_ ← *w*_*j*_,_*i*_ + Δ*w*_*j,i*_ depending on the time difference between the pre- and post-synaptic spikes according to the following equation:


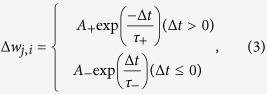



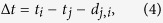


where *t*_*i*_ and *t*_*j*_ are the spike times of neurons *i* and *j*, respectively, and *d*_*j,i*_ is the axonal conduction delay. The parameters *A*_+_, _*−*_ and *τ*_+_, _*−*_ determine the magnitude and duration of potentiation or depression, respectively. We set the parameters such that depression was stronger than potentiation and that the synaptic weight slowly approached zero when the pre-/post-synaptic neurons fired in an uncorrelated manner as follows: *A*_+_ = 0.001, *A*_*−*_ = −0.0012, and *τ*_+_ = *τ*_*−*_ = 20 ms. We decreased the parameters *A*_+__,__*−*_ by a factor of 0.9 every 100 sec. The synaptic weight *w*_*j, i*_ was bounded in the range 0 ≤ *w*_*j,i*_ ≤ *w*_max_, with *w*_max_ = 0.21. The other connections in the model had no plasticity.

We did not model the subcortical structures or detailed microscopic anatomical features such as the six-layered columnar cortical structure. We also did not model any developmental changes with the exception of the activity-dependent plasticity resulting from STDP.

### Sensory feedback to the cortical model

We input proprioception and tactile feedbacks to Brodmann’s areas 3a and 3b in the post-central gyrus and visual feedbacks to the primary visual cortex. We manually selected the target voxels corresponding to these areas and randomly selected neurons receiving sensory feedbacks with a constant density (4 neurons/voxel). Consequently, the number of sensory feedbacks for tactile perception, proprioception, and vision were 912, 376 and 512, respectively (a total of 1,800). As visual feedback, we used 16 × 16 grey scale images for each side captured by a camera fixed in the head. We sequentially input these data to the cortical neurons based on the retinotopic map. We subdivided the somatosensory feedbacks into four groups according to the body parts and randomly input the cortical regions from medial to lateral: leg, trunk, arm and head. All sensory feedbacks arrived as rate codes, which represented the mean firing rates of spiking. To simulate learning in spiking neural networks, a process that exploits precise spike timings between neurons, we converted the rates to spike-timing codes before inputting the cortical neurons using phase-of-firing coding^58^, which has been supported experimentally *in vivo* and *in vitro*^59^.

### Analysis of intrinsic cortical activities

For all analyses of the properties of firing activities, we randomly chose one cortical area and analysed its neurons. We built the resting state functional connectivity based on Pearson’s correlations between the time series of the average firing rates in all possible pairs of 78 cortical regions. We then converted these values to binarized functional connectivity with the same number of edges as the structural connectivity and compared the functional and structural connectivity. To investigate the impact of an extra spike on the cortical network states, we compressed the spike trains using principal component analysis as described in a previous study^60^ (Supplementary Fig. 3e).

### Learning experiments

We set the time step of the computer simulation to 1 ms and performed the learning simulation for 1,000 s. When we compared the sensory response properties between the learned models in the intrauterine and extrauterine conditions, we used the same number of spike trains as inputs by extracting them from the sensory feedbacks in each learning condition. We performed five simulations under each condition (intrauterine and extrauterine) and confirmed the consistency of the results.

## Additional Information

**How to cite this article**: Yamada, Y. *et al*. An Embodied Brain Model of the Human Foetus. *Sci. Rep*. **6**, 27893; doi: 10.1038/srep27893 (2016).

## Supplementary Material

Supplementary Information

Supplementary Video S1
