# Supplementary material for: An Embodied Brain Model of the Human Foetus
Source: Sci Rep. 2016 Jun 15;6:27893. doi: 10.1038/srep27893 (PMC4908381; doi:10.1038/srep27893)
Supplement: Supplementary Information [file srep27893-s1.pdf]

Supplementary Information

Title:

An Embodied Brain Model of the Human Foetus

Authors:

Yasunori Yamada[1,2*†], Hoshinori Kanazawa[1,2], Sho Iwasaki[1], Yuki Tsukahara[1], Osuke Iwata[3], Shigehito Yamada[4], and Yasuo Kuniyoshi[1*]

Affiliations:

[1]Graduate School of Information Science and Technology, The University of Tokyo, Tokyo 113-8656, Japan.

[2]Research Fellow of the Japan Society for the Promotion of Science.

[3]Department of Paediatrics and Child Health, Kurume University School of Medicine, Fukuoka 830-0011, Japan.

[4]Congenital Anomaly Research Center, Kyoto University Graduate School of Medicine, Kyoto 606-8501, Japan.

†Present address: IBM Research-Tokyo, Hakozaki Office, 19-21 Nihonbashi Hakozaki-cho Chuo-ku Tokyo, 103-8510 Japan.

*Corresponding author.

E-mail: y-yamada@isi.imi.i.u-tokyo.ac.jp, kuniyosh@isi.imi.i.u-tokyo.ac.jp

Supplementary Methods

Musculoskeletal body model. We constructed the bone, muscle and skin of the foetus model mainly based on foetal MRI data and used other complementary data when necessary because of the limited resolution of the MRI data. The human foetus examined by MRI was a historical specimen with a gestational age of 206 days belonging to the Kyoto Collection[1]. The scan was conducted using a 1.5 T MRI system (Excelart Vantage, Toshiba Medical Systems, Tokyo, Japan). We used T1-weighted images and a 3D gradient-echo sequence with the following parameters: a time repetition/time echo of 30/7 ms, an imaging matrix of 256×192×176 pixels, and a spatial resolution of 0.61×0.61×0.80 mm. We obtained the approval of the local ethical committees of Kyoto University and The University of Tokyo, and the procedures were performed in accordance with the Ethical Guidelines for Medical and Health Research Involving Human Subjects. From the MRI data, we automatically segmented the skin and manually segmented the bones and muscles.

For the skeletal model, we additionally used a foetal skeleton replica corresponding to 32 gestational weeks from Bone Clones, Inc. (http://www.boneclones.com/) and acquired computed tomography scans with a spatial resolution of 0.02 mm. The MRI provided information on the global bone shape, including cartilage, although we could not obtain detailed structures of some features, such as the bones of the hands and skull owing to resolution limitations. In contrast, the data extracted from the foetal skeleton replica had high spatial resolution, but little cartilage. We constructed the foetal skeletal model by combining both data sets using MAYA (Autodesk). Finally, we confirmed that the bone sizes were within the normal range of human foetuses at 32 gestational weeks with respect to the following parameters: biparietal diameter, head circumference, humerus, radius, ulna, femur, tibia and fibula[2, 3].

We modelled 830 muscles as piecewise line segments defined by attachment and relay points based on Lee's work[4] and built the foetal musculoskeletal body model by combining the foetal MRI data and an adult musculoskeletal model[4]. We extracted the attachment and relay points of 216 muscles from the foetal MRI data. We obtained those

of the other muscles by manually transforming the adult bones to foetal bones using MAYA. We also calculated the muscle cross-sectional area of the 216 muscles extracted from the foetal MRI data to estimate the maximal voluntary contraction force[5]. We calculated the cross-sectional areas of the muscles that could not be extracted from the foetal MRI data by scaling the adult data using the average ratio calculated using the extracted foetal muscles. Because we subdivided the body model into 21 rigid-body parts for physical simulation, we excluded all muscles whose attachment and relay points belong to only one rigid-body and ultimately adapted 390 muscles in the musculoskeletal body model (Fig. 1b and Supplementary Table 1).

A total of 20 joints with 36 degrees of freedom were modelled in the entire body by excluding the fingers and toes and simplifying the vertebrae. The range of motion of each joint was set such that it did not exceed the human neonate data for the limbs[6] or the adult data for the trunk, neck and scapula[7], which were used because no neonatal data were available for these three factors.

To simulate the three-dimensional rigid-body dynamics, we used the Open Dynamics Engine, which is a widely used open-source physics engine (http://www.ode.org/). Muscle dynamics and proprioceptive sensory feedback for the muscle spindle and Golgi tendon organ were modelled based on experimental data[8]. The dynamics in the original models were defined for one specific muscle; thus, we applied them to the muscles in the entire body by normalising the maximal force, length and range. We allowed the foetal model to generate random movements within the range of motion of the joints and determined the range of lengths of each muscle to normalize the sensitivity of the muscle spindle model.

The data for the model of the foetal musculoskeletal body will be posted on the website of the Intelligent Systems & Informatics Laboratory at The University of Tokyo (http://www.isi.imi.i.u-tokyo.ac.jp/public/foetal_model).

Spinal neural circuit model. We employed the spinal neural circuit model[9] (Fig. 1g), which is based on experimental data and is scalable for the whole-body musculoskeletal model[10, 11]. The spinal circuit model independently controls each muscle within an elementary circuit consisting of the following components: neural oscillator, sensory interneuron, and α and γ motor neurons. The spinal circuit relays sensory feedback from the muscle spindle and tactile mechanoreceptor models to cortical neurons via sensory interneurons and modulates the muscle activation and sensitivity of the spindle with basic neuromuscular loops, such as the stretch reflex and Alpha-Gamma linkage. The neural oscillators are considered the neural bases of spontaneous movements[12]. These spontaneous movements can be observed as early as muscles function[13], and many animal studies suggest that their essential role in early nervous and motor development begins in the foetal period[14, 15, 16]. However, the spinal neural circuit that generates whole-body movements is still unknown. Therefore, we built a minimal spinal circuit model based on experimentally supported interneuronal connectivity and investigated whether the simulated movements could capture the normal features that have been reported in human studies.

The dynamics of the neural oscillator model are represented by the Bonhoffer-van der Pol (BVP, or FitzHugh-Nagumo) equation as follows[9, 17]:

$$\tau \frac{\mathrm{d}x}{\mathrm{d}t} = \mathrm{c}\left(\mathrm{x} - \frac{1}{3}x^3 - y + I_\mathrm{c}\right) + \delta(I_s - x), \tag{1}$$

$$\tau \frac{\mathrm{d}y}{\mathrm{d}t} = \frac{1}{c}(\mathrm{x} - by + a) + \epsilon I_\mathrm{c}, \tag{2}$$

where I_c and I_s are the inputs from the cortex and spinal sensory interneurons, respectively. The corticospinal input is modelled as a constant value, I_c = 0.6, because the mechanism by which cortical learning alters generated movements is outside the scope of this paper and because the functional development of corticospinal tracts, such as the transmission of motor signals, is not thought to begin during the early periods of cortical learning targeted in this paper[18]. The other constant parameters were set as a = 0.7, b = 0.675, c = 1.75, δ = 0.013, ϵ = 0.022, and τ = 0.2 based on previous work[9]. The sensory interneurons and α and γ motor neurons were modelled using the following transfer functions based on experimental data[8]:

$$\frac{m(s)}{i(s)} = \frac{k_\mathrm{m}(1 + s/33 + (s/33)^2)}{1 + 2(s/58) + (s/58)^2}, \tag{3}$$

where k_m was set to 1.5 p/s/nA[19]. For more details, see the original paper[9].

Intrauterine and extrauterine environment models. In the intrauterine environmental condition, the centre of the uterus was spatially fixed and the centre of the abdomen of the foetal model was connected to the centre of the uterus by a ball joint. Thus, the abdomen body part could rotate only around its centre. In the intrauterine condition, the foetal model received forces from the uterine membrane, amniotic fluid and physical contact between body parts in addition to gravity and buoyancy forces. The forces from the uterine membrane and amniotic fluid were calculated for each point on the skin surface.

To simulate the extrauterine environmental condition, we placed the body model on a flat, rigid bed. The model was subject to only the forces of gravity and physical contact between body parts and the flat, rigid bed.

In both conditions, we set the acceleration of gravity to 9.8 m/s^2. The forces due to the physical contact were calculated by the physical simulator.

If $\boldsymbol{F}_{\mathrm{u},i}$ denotes the force from the uterine membrane at point i on the skin surface of body part j, then $\boldsymbol{F}_{\mathrm{u},i}$ is calculated as follows:

$$\boldsymbol{F}_{\mathrm{u},i} = -f_{\mathrm{u},i} A_i \boldsymbol{n}_i, \tag{4}$$

$$f_{\mathrm{u},i} = \begin{cases} (K_\mathrm{u} r_i + C_\mathrm{u} \dot{r}_i)_+ \dfrac{(\boldsymbol{n}_i \cdot \boldsymbol{q}_i)_+}{\|\boldsymbol{q}_i\|} & (r_i > 0) \\ 0 & (r_i \le 0) \end{cases}, \tag{5}$$

$$r_i = \|\boldsymbol{q}_i\| - R_\mathrm{u}, \tag{6}$$

$$\boldsymbol{q}_i = \boldsymbol{p}_i - \boldsymbol{p}_\mathrm{u}, \tag{7}$$

$$A_i = A_j \frac{a_i}{\sum_n a_n}, \tag{8}$$

where $\boldsymbol{p}_i$ and $\boldsymbol{n}_i$ are the position and normal vectors, respectively. $\boldsymbol{p}_\mathrm{u}$ is the position of the centre of uterine membrane and R_u is the minimum radius at which the force occurs. K_u and C_u are the viscoelastic coefficients. We set R_u = 0.05 m, K_u = 1.0 Pa/m and C_u = 0.01 Pa·s/m. $(x)_+$ indicates rectification, and any value of $x \leq 0$ is 0. A_j and a_i are the skin surface areas of body part j and the polygon mesh at i, respectively.

We defined the force caused by amniotic fluid resistance $\boldsymbol{F}_{\mathrm{a},i}$ as follows:

$$\boldsymbol{F}_{\mathrm{a},i} = -f_{\mathrm{a},i} A_i \boldsymbol{n}_i, \tag{9}$$

$$f_{\mathrm{a},i} = \frac{1}{2} \rho_\mathrm{a} C_\mathrm{D} (\boldsymbol{v} \cdot \boldsymbol{n}_i)^2 . \tag{10}$$

Here, $\boldsymbol{v}$ denotes the velocity of the body part, ρ_a is the density of the amniotic fluid and C_D is a drag coefficient. We set ρ_a = 1,010 kg/m^3 based on published data[20] and C_D = 1.0 Pa·m/kg·s^2.

In the intrauterine condition, we also simulated buoyancy forces using the densities of the amniotic fluid ρ_a and the foetal body model ρ_b. We set ρ_b = 1,020 kg/m^3.

Tactile model. To simulate tactile sensation in our rigid-body model, we modelled Merkel cells, which are mechanoreceptors that mainly detect continuous pressure. The Merkel cells were modelled as low-pass filters of the pressure inputs (< 50 Hz)[21, 22]. The inputs of the Merkel cell model consisted of three types of pressure inputs resulting from the interactions with the uterine membrane $f_{\mathrm{u},i}$, amniotic fluid resistance $f_{\mathrm{a},i}$ and physical contact with the uterine environment. In the extrauterine environmental condition, the input of the Merkel cell model included only the pressure due to physical contact. The pressure exerted by physical contact of each tactile point was determined by distributing a physical contact force calculated by the physical simulator. When object j with tactile point i contacts object k, $\boldsymbol{F}_{\mathrm{p},jk}$ denotes the contact force calculated by the

physical simulator. We distributed $\boldsymbol{F}_{\mathrm{p},jk}$ to tactile point i as $f_{\mathrm{p},i}$ as described in the following equation:

$$f_{\mathrm{p},i} = \frac{1}{A_i} \frac{d_{i,k}}{\sum_n d_{n,k}} \left\| \boldsymbol{F}_{\mathrm{p},jk} \right\|, \tag{11}$$

$$d_{i,k} = A_i (L_\mathrm{c} - l_{i,k})_+ (-\boldsymbol{F}_{\mathrm{p},jk} \cdot \boldsymbol{n}_i)_+ \, . \tag{12}$$

Here, $l_{i,k}$ is the distance to object k along a normal vector $\boldsymbol{n}_i$. L_c is the minimum distance to which the physical contact force is distributed, and we set L_c = 5 mm. The 3,000 tactile mechanoreceptor models were distributed according to human two-point discrimination data[23] (Fig. 1d and Supplementary Table 2).

Analysis of whole-body movements. Regarding the chaotic and fractal properties of whole-body movements, we simulated foetal movements for 1,000 s and then analysed the trajectories of the four limbs based on experimental studies of human neonates and infants. To investigate the chaotic properties, we calculated the largest Lyapunov exponent[24]. When the exponent was positive, the trajectory exhibited characteristics of deterministic chaotic properties. We quantified the fractal properties by calculating the scaling exponent using detrended fluctuation analysis[25]. When the exponent was close to one, the trajectory exhibited fractal-type, long-range correlations. To investigate whether the generated whole-body movements were well-coordinated, a qualitative feature reported in human studies[26, 27], we analysed inter-limb coordination by measuring the degree of phase synchronization between muscles using the standard shuffle-corrected phase synchronization index[28].

Supplementary Results

Intrinsic cortical activities after learning. We investigated the intrinsic activities of the cortical models after learning in the intrauterine and extrauterine conditions. We found that both cortical models exhibited self-sustained activities that included the following distinctive properties observed in physiological cortical activity:

(1) low firing rates of individual excitatory neurons (intrauterine: 2.5 Hz; extrauterine: 2.7 Hz) with distributions that approximated log-normal distributions (intrauterine: R^2 = 0.99 for excitatory neurons, R^2 = 0.96 for inhibitory neurons, $P < 10^{-20}$; extrauterine: R^2

= 0.99 for excitatory neurons, R^2 = 0.99 for inhibitory neurons, $P < 10^{-20}$);
(2) irregular neuronal firing following a Poisson distribution (Intrauterine: CV = 1.07 for excitatory neurons, CV = 1.06 for inhibitory neurons; Extrauterine: CV = 1.08 for excitatory neurons, CV = 1.07 for inhibitory neurons; CV = coefficient of variation of the inter-spike intervals);
(3) a network balance between excitation and inhibition (Intrauterine: r = 0.88, $P < 10^{-20}$; Extrauterine: r = 0.89, $P < 10^{-20}$);
(4) greater depolarizations of the average membrane potentials relative to the resting potentials (Intrauterine: -63.9 mV for excitatory neurons, -56.0 mV for inhibitory neurons; Extrauterine: -64.6 mV for excitatory neurons, -55.6 mV for inhibitory neurons);
(5) a correlation between the structural and functional connectivity across the cortical regions (Intrauterine: r = 0.78; $P < 10^{-9}$; Extrauterine: r = 0.79; $P < 10^{-9}$); and
(6) responsiveness to single spikes (the time required to completely change the state of the entire cortex was 240 ms and 230 ms for the cortical models learned in the intrauterine and extrauterine conditions, respectively).
In terms of these six features, we confirmed that the learned cortical model maintained the biological relevance of the intrinsic cortical activities.

Supplementary Table 1 | List of the muscles modelled in the foetal body. A total of 390 muscles were modelled. The numbers indicate the number of multiple line segments of each muscle model.

Head/Neck muscles (76)
Longissimus capitis (16), Splenius capitis (10), Semispinalis capitis (18), Longus capitis (8), Obliquus capitis (2), Rectus capitis posterior (4), Rectus capitis anterior (2), Scalenes (10), Sternocleidomastoid (4), Rectus capitis lateralis (2)
Trunk muscles (156)
Rectus abdominis (2), External internal obliques (22), Iliocostalis lumborum (24), Spinalis thoracis (8), Trapezius (12), Pectoralis minor (6), Serratus anterior (16), Subclavius (2), Longissimus thoracis (48), Serratus posterior inferior (4), Rhomboid major (2), Quadratus lumborum (10)
Arm muscles (37×2 = 74)
Pectoralis major (6), Latissimus dorsi (7), Biceps brachii (2), Brachioradialis, Brachialis, Coracobrachialis, Triceps brachii (3), Infraspinatus, Deltoid (2), Supraspinatus, Teres major, Extensor carpi radialis brevis, Extensor carpi ulnaris, Extensor carpi radialis longus, Flexor carpi ulnaris, Palmaris longus, Flexor digitorum superficialis (3), Pronator teres, Flexor carpi radialis, Pronator quadratus
Leg muscles (42× 2 = 84)
Gluteus Maximus (3), Gemellus (2), Piriformis, Obturator (2), Rectus femoris, Sartorius, Quadratus femoris, Adductor brevis, Adductor longus, Gracilis, Adductor magnus (2), Extensor digitorum longus, Peroneus brevis, Peroneus longus, Peroneus tertius, Vastus, Semimembranosus, Semitendinosus, Pectineus, Iliopsoas (6), Soleus, Extensor hallucis longus, Flexor hallucis longus, Flexor digitorum longus, Gastrocnemius (2), Plantaris, Popliteus, Biceps femoris (2), Tensor fascia latae, Tibialis anterior

Supplementary Table 2 | Tactile distribution in the foetus model. A total of 3,000 tactile sensors were allocated based on the two point discrimination (TPD) described by Weinstein [23].

Body	TPD (mm)	Tactile sensors
Head	17.8	1,286
Neck	31.7	37
Chest	36.5	155
Abdomen	36.5	102
Hip	36.5	100
Upper arm	46.2	62
Forearm	39.0	74
Hand	10.5	802
Thigh	45.2	92
Calf	48.0	48
Foot	21.6	242

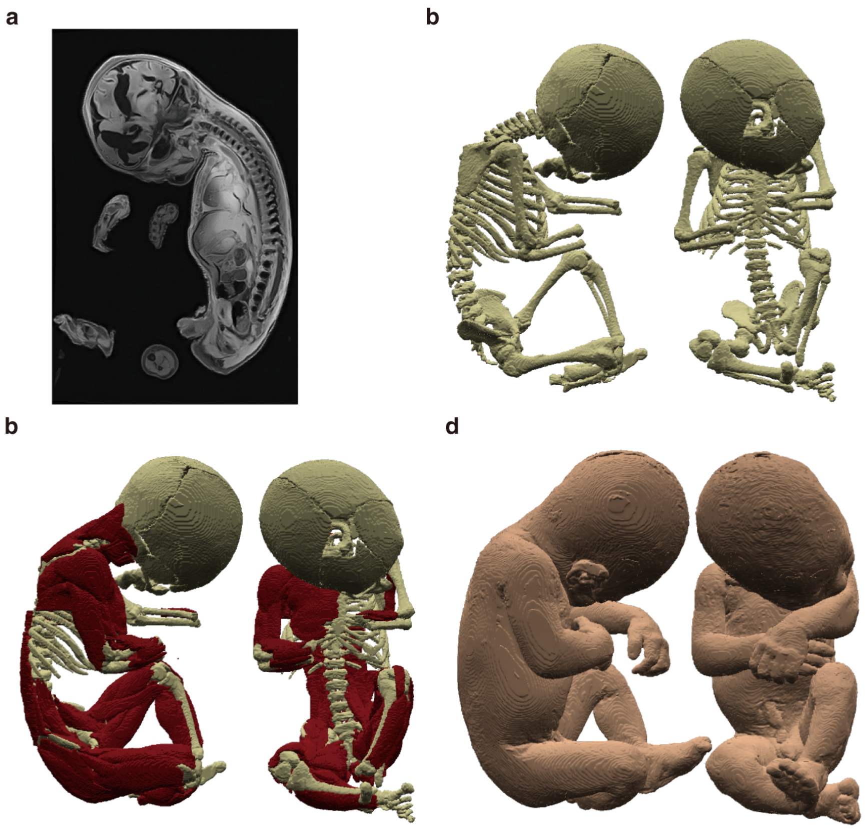

Supplementary Figure 1 | Foetal model extracted from the MR images. (a) MR images of the foetal specimen with a menstrual age of 206 days. **(b, c, and d)** Foetal skeleton, muscles, and skin extracted from the MR images.

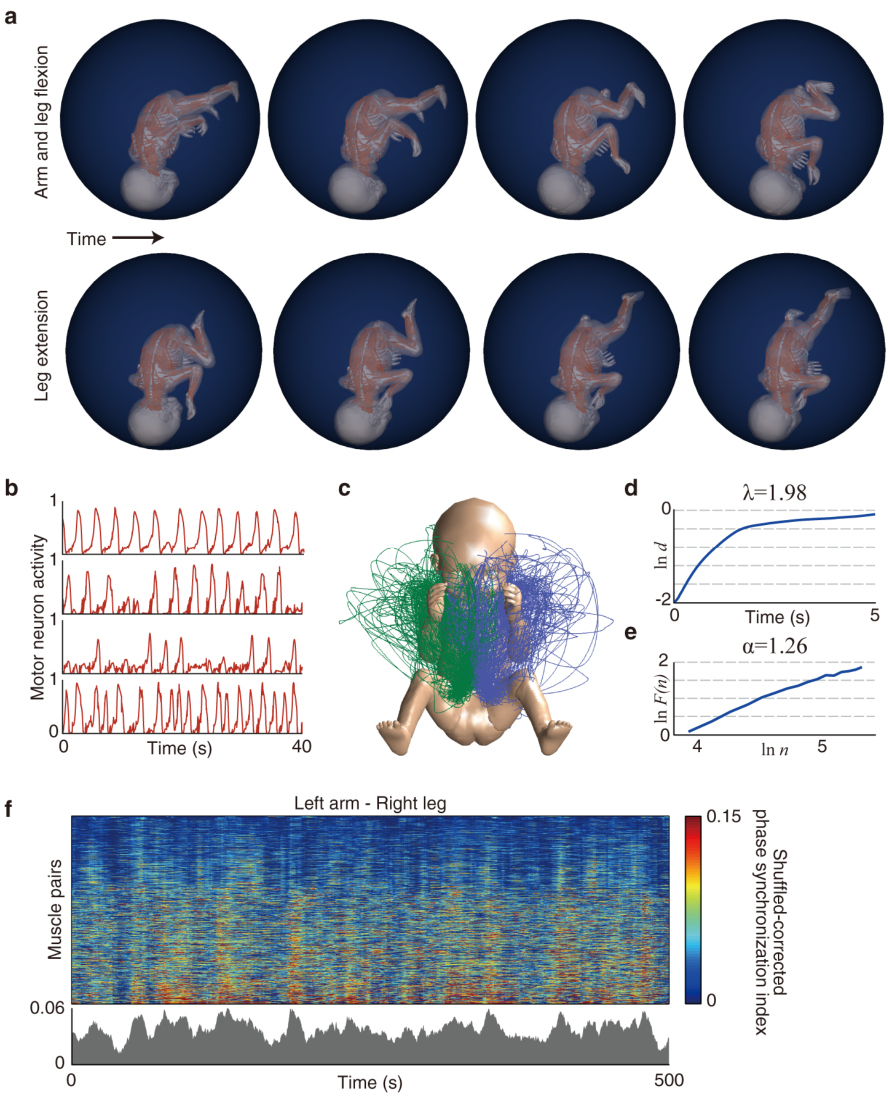

Supplementary Figure 2 | Simulated whole-body movements and their characteristics. (a) Snapshots of simulated movements. We confirmed that the simulated movements involved the participation of multiple body parts and continuously varying combinations of body part movement directions. **(b)** Examples of a time series of α motor neuron outputs. **(c)** Trajectories of the hands. **(d)** Time evolution of the log-transformed divergence used to calculate the maximal Lyapunov exponents λ of the left hand trajectory. The maximal Lyapunov exponents of the four limbs ranged from 1.98 to 2.42, indicating that the trajectories of the limbs exhibited characteristics of deterministic chaotic properties. **(e)** Detrended fluctuation analysis of the left hand trajectory. The calculated scaling exponents α of the four limbs ranged from 1.15 to 1.28, which are similar to the values reported in human studies [29] and show fractal-type, long-range

correlations in the limb trajectories. **(f)** Time evolution of the shuffled-corrected phase synchronization indices of all possible pairs of muscles between the left arm and right leg. The bottom plot shows the change in its average value. The phase synchronization patterns dynamically emerge and continuously change, which was observed in all limb pairs.

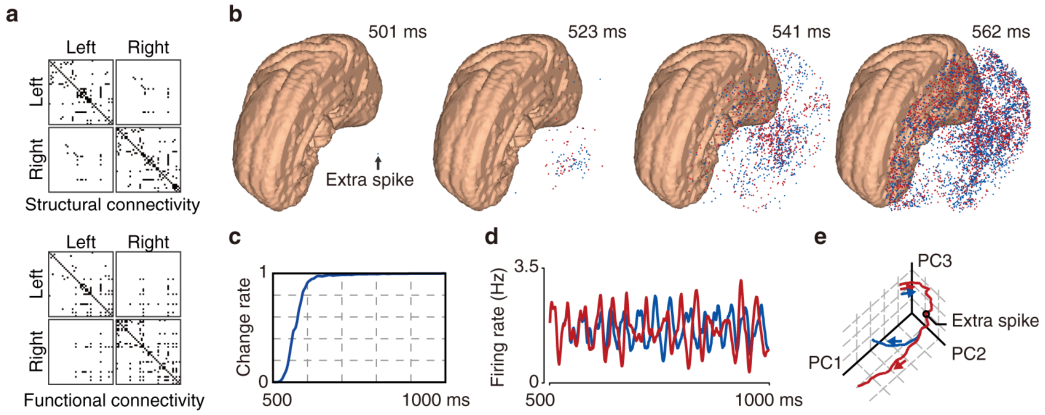

Supplementary Figure 3 | Global features of the simulated intrinsic cortical activity. (a) Structural and functional connectivities between 78 cortical regions. Both topologies are correlated in terms of network degree ($r = 0.74$; $P < 10^{-9}$). **(b, c, d and e)** Impact of a single additional spike on all cortical activities. **(b)** Differences in brain activities with and without the extra spike at 501 ms. Each dot denotes a spike generated (blue) or extinguished (red) by the extra spike. **(c)** Time evolution of the ratio of the number of extra or missing spikes to all spikes throughout the brain. Approximately 310 ms were required to completely change the state of the entire cortex. **(d)** Average firing rates of the two simulations in the brain area with the extra spike. **(e)** Time evolution in the 3 principal components of neural activities.

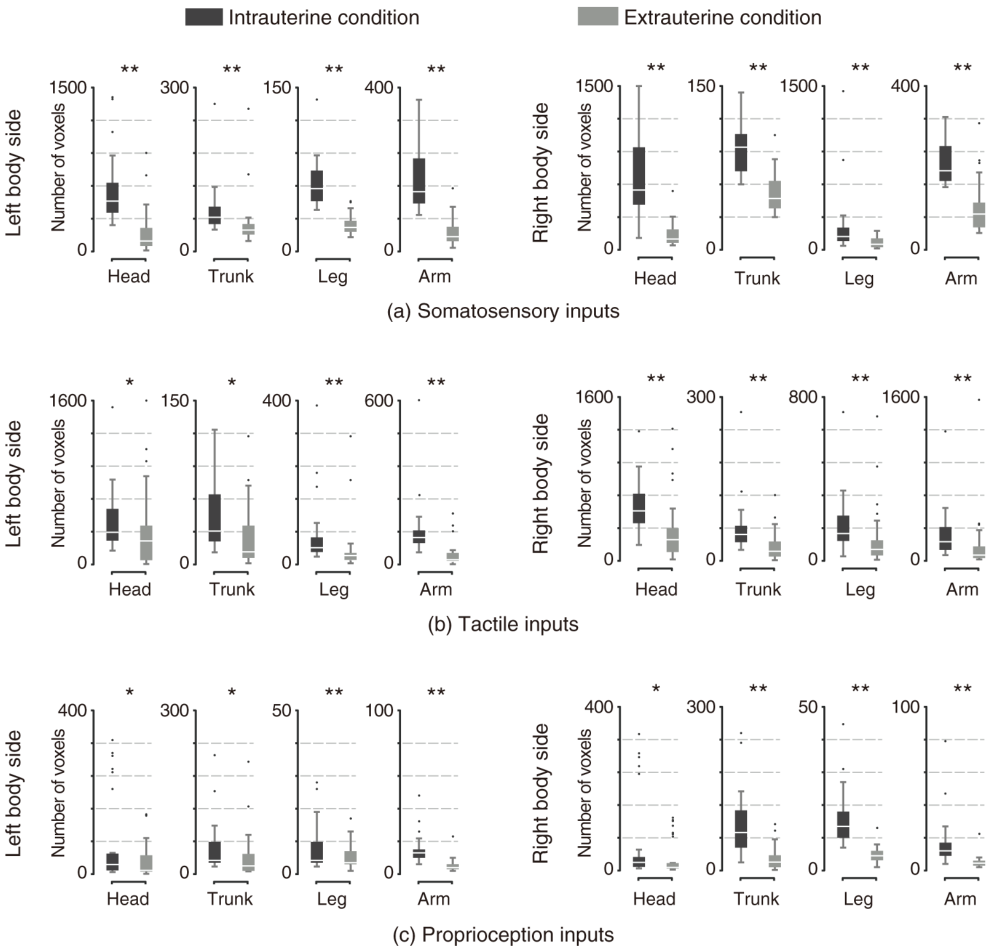

Supplementary Figure 4 | Cortical regions that significantly respond to sensory feedbacks in the learned cortex models under the intrauterine and extrauterine environmental conditions. In all tested body parts, the number of these cortical regions significantly increased in the learned cortex models inside the uterus (*P < 0.05, **P < 10^{-3}, rank-sum test). Somatosensory inputs consisted of tactile and proprioception inputs. The whiskers in the box plots indicate the upper and lower quartiles.

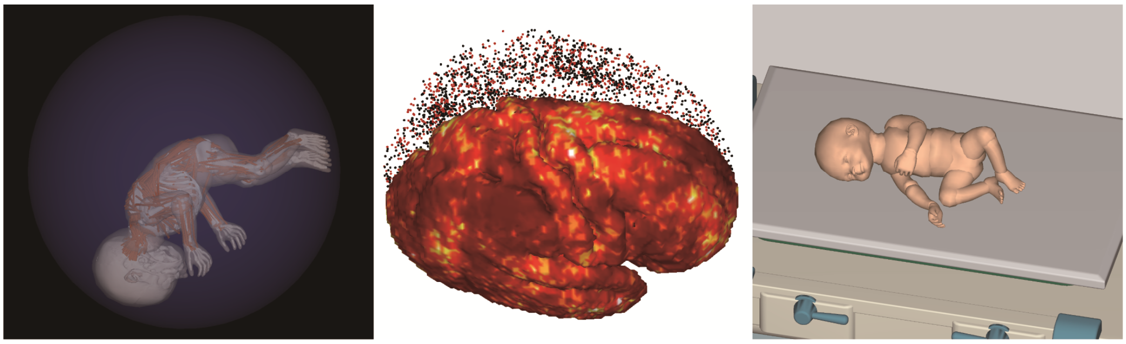

Supplementary Video 1 | Embodied brain model of a human foetus. The video shows examples of the simulated whole-body movements in the intrauterine environmental condition and the intrinsic cortical activity and whole-body movements in the extrauterine environmental condition.

References

1. Kameda T, Yamada S, Uwabe C, Suganuma N. Digitization of clinical and epidemiological data from the Kyoto Collection of Human Embryos: maternal risk factors and embryonic malformations. *Congenital anomalies* **52**, 48-54 (2012).

2. Archie JG, Collins JS, Lebel RR. Quantitative standards for fetal and neonatal autopsy. *Am. J. Clin. Pathol.* **126**, 256-265 (2006).

3. Chitty LS, Altman DG. Charts of fetal size: limb bones. *BJOG* **109**, 919-929 (2002).

4. Lee SH. Biomechanical modeling and control of the human body for computer animation. *Computer Science Department* **Ph.D. dissertation**, 143 (2008).

5. Maughan R, Watson J, Weir J. Strength and cross‐sectional area of human skeletal muscle. *J. Physiol.* **338**, 37-49 (1983).

6. Watanabe H, Ogata K, Amano T, Okabe T. [The range of joint motions of the extremities in healthy Japanese people--the difference according to the age (author's transl)]. *Nihon Seikeigeka Gakkai zasshi* **53**, 275-261 (1979).

7. Yonemoto K, Ishigami S, Kondoh T. Kansestukadouikihyouji narabini sokuteihou (*in Japanese*). *The Japanese Association of Rehabilitation Medicine* **32**, 207-217 (1995).

8. He J, Maltenfort MG, Wang Q, Hamm TM. Learning from biological systems: Modeling neural control. *Control. Syst. Mag.* **21**, 55-69 (2001).

9. Kuniyoshi Y, Sangawa S. Early motor development from partially ordered neural-body dynamics: experiments with a cortico-spinal-musculo-skeletal model. *Biol. Cybern.* **95**, 589-605 (2006).

10. Yamada Y, Kuniyoshi Y. Emergent Spontaneous Movements Based on Embodiment: Toward a General Principle for Early Development. *Post-Graduate Conference on Robotics and Development of Cognition*, 52-55 (2012).

11. Yamada Y, Nishikawa S, Shida K, Niiyama R, Kuniyoshi Y. Neural-body coupling for emergent locomotion: A musculoskeletal quadruped robot with spinobulbar model.

IEEE/RSJ Int. Conf. Robot. Syst., 1499-1506 (2011).

12. Hadders-Algra M. Putative neural substrate of normal and abnormal general movements. *Neurosci. Biobehav. Rev.* **31**, 1181-1190 (2007).

13. Lüchinger AB, Hadders-Algra M, Van Kan CM, de Vries JI. Fetal onset of general movements. *Pediatr. Res.* **63**, 191-195 (2008).

14. Granmo M, Petersson P, Schouenborg J. Action-based body maps in the spinal cord emerge from a transitory floating organization. *J. Neurosci.* **28**, 5494-5503 (2008).

15. Petersson P, Waldenström A, Fåhraeus C, Schouenborg J. Spontaneous muscle twitches during sleep guide spinal self-organization. *Nature* **424**, 72-75 (2003).

16. Khazipov R, Sirota A, Leinekugel X, Holmes GL, Ben-Ari Y, Buzsáki G. Early motor activity drives spindle bursts in the developing somatosensory cortex. *Nature* **432**, 758-761 (2004).

17. Asai Y, Nomura T, Sato S. Emergence of oscillations in a model of weakly coupled two Bonhoeffer–van der Pol equations. *Biosystems* **58**, 239-247 (2000).

18. Martin JH. The corticospinal system: from development to motor control. *The Neuroscientist* **11**, 161-173 (2005).

19. Baldissera F, Campadelli P, Piccinelli L. The dynamic response of cat α-motoneurones investigated by intracellular injection of sinusoidal currents. *Exp. Brain Res.* **54**, 275-282 (1984).

20. Hsu C-D*, et al.* Elevated amniotic fluid levels of leukemia inhibitory factor, interleukin 6, and interleukin 8 in intra-amniotic infection. *Am. J. Obstet. Gynecol.* **179**, 1267-1270 (1998).

21. Shirado H, Konyo M, Maeno T. Modeling of tactile texture recognition mechanism. *Journal of the Japan Society of Mechanical Engineers* **73**, 2514-2522 (2007).

22. Freeman AW, Johnson KO. A model accounting for effects of vibratory amplitude on

responses of cutaneous mechanoreceptors in macaque monkey. *J. Physiol.* **323**, 43-64 (1982).

23. Weinstein S. Intensive and extensive aspects of tactile sensitivity as a function of body part, sex and laterality. *on the Skin Senses*, (1968).

24. Rosenstein MT, Collins JJ, De Luca CJ. A practical method for calculating largest Lyapunov exponents from small data sets. *Physica D* **65**, 117-134 (1993).

25. Peng CK, Havlin S, Stanley HE, Goldberger AL. Quantification of scaling exponents and crossover phenomena in nonstationary heartbeat time series. *Chaos* **5**, 82-87 (1995).

26. Prechtl HFR. Qualitative changes of spontaneous movements in fetus and preterm infant are a marker of neurological dysfunction. *Early Hum. Dev.* **23**, 151-158 (1990).

27. Prechtl HFR. General movement assessment as a method of developmental neurology: new paradigms and their consequences. *Dev. Med. Child Neurol.* **43**, 836-842 (2001).

28. Tass P*, et al.* Detection of n: m phase locking from noisy data: application to magnetoencephalography. *Phys. Rev. Lett.* **81**, 3291 (1998).

29. Waldmeier S*, et al.* Correlation properties of spontaneous motor activity in healthy infants: a new computer-assisted method to evaluate neurological maturation. *Exp. Brain Res.* **227**, 433-446 (2013).
